# Supplementary material for: Using experience to improve: how errors shape behavior and brain activity in monkeys
Source: PeerJ. 2018 Jul 27;6:e5395. doi: 10.7717/peerj.5395 (PMC6065460; doi:10.7717/peerj.5395)
Supplement: Supplemental Information 1 [file peerj-06-5395-s001.zip › AssociatedData_PardoVazquezAndAcuña_PeerJ.pdf]

Associated data description

Title: Using experience to improve: How errors shape behavior and brain activity in monkeys

Authors: Jose L. Pardo-Vazquez and Carlos Acuña

Associated data includes both psychophysical and electrophysiological data (single cells) obtained from one monkey performing the length discrimination task described in the ms.

- Psychophysical data can be found in one Matlab file (Psychophysics_PeerJ.mat), which includes three vectors in which each element is a trial:
    - Condition_fTrial, coded as two digits. The first digit corresponds to the length of the S1 (1 for 2º, 2 for 2.18º and 3 for 2.36º), the second digit corresponds to the relation between the S2 and the S1 (1, S2<S1, easy; 2, S2<S1, difficult; 3, S2>S1, difficult; and 4, S2>S1, easy).
    - Outcome_fTrial, coded as one digit (0 for error, 1 for correct, and 2 for abort).
    - RT_fTrial, reaction time.

- Electrophysiological data can be found in one compressed folder (Neurons_PeerJ). The folder includes 686 files, one for each neuron we were able to isolate during the recordings. Each file includes 4 elements:
    - FiringRate_fTrial. Each row is a time bin and each column a trial. Firing rates were estimated using a 10 ms sliding window.
    - timeBins, which is the timing of the firing rates
    - Markers_Labels_fTrial. Each column includes the sequence of event markers for each trial. The meaning of the markers is briefly described below:
        - NT, New trial
        - Condition code:
            - First digit (S1 length):
                - 1 – 2º
                - 2 – 2.18º
                - 3 – 2.36º
            - Second digit (sign(S2-S1) and difficulty)
                - 1 – S2<S1, Easy
                - 2 – S2<S1, Difficult
                - 3 – S2>S1, Difficult
                - 4 – S2>S1, Easy
        - FX, fixation target onset
        - S1, S1 onset
        - De, delay onset
        - S2, S2 onset
        - CB, capture choice button
        - RI/RC, incorrect response/correct response
        - RW, reward (missing for incorrect responses)
        - ET, End trial
    - Markers_TimeStamps_fTrial. Timestamps (from the beginning of the recording) for each event marker included in the Matlab cell Markers_Labels_fTrial
